# Green Synthesis of ZnO Nanoparticles Using *Ocimum basilicum* var. *purpurascens*: As-Synthesized Phase Formation and Thermal Evolution of Optical Properties

**DOI:** 10.3390/nano16100572

**Published:** 2026-05-07

**Authors:** Jorge L. Iriqui-Razcón, José L. De-la-Cruz-Estrella, Francisco Brown-Bojórquez, Hiram J. Higuera-Valenzuela, Pedro A. Hernández-Abril, Jesús A. Maldonado-Arriola, José A. Heredia-Cancino

**Affiliations:** 1 Ingeniería Biomédica, Unidad Académica Hermosillo, Universidad Estatal de Sonora, Ley Federal del Trabajo s/n, Col. Apolo, Hermosillo 83100, Mexico; jorge.iriqui@ues.mx (J.L.I.-R.); hiram.higuera@ues.mx (H.J.H.-V.); pedro.hernandez@ues.mx (P.A.H.-A.); jesusantonio.maldonado@unison.mx (J.A.M.-A.); 2 Maestría en Ciencia de Materiales, Unidad Académica Hermosillo, Universidad Estatal de Sonora, Ley Federal del Trabajo s/n, Col. Apolo, Hermosillo 83100, Mexico; 3 Departamento de Investigación en Polímeros y Materiales, Universidad de Sonora (UNISON), Hermosillo 83000, Mexico; francisco.brown@unison.mx; 4 Departamento de Ingeniería Industrial, Universidad de Sonora (UNISON), Hermosillo 83000, Mexico

**Keywords:** Zinc oxide, nanoparticles, green synthesis, thermal annealing, band gap, *Ocimum basilicum*, structural evolution

## Abstract

This study reports a facile, phyto-mediated synthesis of ZnO nanoparticles utilizing the *Ocimum basilicum* var. *purpurascens* extract. The high phenolic (1807.28 ± 57.38 µmol GAE/g) and flavonoid (33.17 ± 3.50 µmol QE/g) contents of the extract successfully induced the formation of the crystalline hexagonal wurtzite phase under mild reaction conditions, circumventing the conventional requirement for the initial high-temperature calcination. The structural, morphological, and optical evolution of the nanoparticles was systematically evaluated from their as-synthesized state to the thermal annealing temperatures of 700 °C to 900 °C. X-ray diffraction analysis confirmed an increase in crystallite size from 21.5 nm to 55.6 nm, while scanning electron microscopy revealed a corresponding growth in average particle size from 143.33 nm to 261.50 nm due to thermal sintering. Furthermore, FTIR and EDS verified the degradation of organic capping agents and a progressive stoichiometric refinement, with the high-temperature samples approaching theoretical elemental purity (18.22% normalized oxygen mass). Optical characterization demonstrated a red-shift in the band gap energy from 3.28 eV to 3.21 eV, alongside a significant increase in visible diffuse reflectance progressing from a baseline of 30–60% to values exceeding 95% upon the removal of the biochemical components. These findings validate the OBPE-mediated protocol as a sustainable, thermodynamically advantageous route for producing structurally and optically tunable ZnO nanomaterials for advanced applications.

## 1. Introduction

Zinc oxide (ZnO) is an established multi-functional material within the class of n-type semiconducting metal oxides [1,2]. Its physicochemical importance stems from its unique intrinsic characteristics, such as a wide direct band gap of approximately 3.37 eV and a substantial exciton binding energy of 60 meV [3,4]. These properties provide ZnO with high chemical stability, a broad absorption spectrum, and strong electrochemical coupling, which facilitates its extensive applications in optoelectronics, sensors, and catalysis [3,5]. Furthermore, ZnO stands out among other metal oxides for its favorable biocompatibility profile; it is listed as a Generally Recognized As Safe (GRAS) material by the US Food and Drug Administration (FDA) [3,5]. Consequently, ZnO nanoparticles (NPs) have attained significant attention in the biomedical field, where their low toxicity, antibacterial, anti-inflammatory, and anticancer activities offer promising advancements for therapeutic applications [6,7].

Despite the functional versatility of ZnO NPs, producing them at an industrial scale via conventional physicochemical routes, such as sol–gel, hydrothermal, and laser ablation, faces significant sustainability challenges due to high energy consumption and the requirement for expensive equipment [7]. Furthermore, these methods often require toxic reducing agents and hazardous organic solvents, generating by-products that may pose environmental risks and potentially induce cytotoxicity in biomedical applications [8,9]. To address these limitations, developments in green synthesis have emerged, offering a cost-effective, eco-friendly, and scalable alternative that reduces the usage of dangerous chemicals [10,11]. For this research, the chemical components provided by the *Ocimum basilicum* var. *Purpurascens* Extract (OBPE) served as a chelating agent and stabilizer for the intermediate zinc ion species. Crucially, these molecules facilitate the stabilization and crystallization of ZnO nanostructures under mild reaction conditions, offering a significant advantage by avoiding the annealing steps typically required in conventional synthesis procedures [10,12].

Among the diverse botanical sources available for green synthesis, *Ocimum basilicum* (Basil) is distinguished by its spectrum of bioactive secondary metabolites, including phenolic acids and flavonoids, which contribute to its pharmacological potential [13,14]. However, the Purpurascens variety (Purple Basil) was selected for this study due to its phytochemical profile, characterized by its high concentrations of compounds such as anthocyanins responsible for its purple pigmentation and rosmarinic acid compared to common green cultivars [15]. These anthocyanins, alongside other antioxidants identified in the extract, exhibit superior redox potential, acting as highly efficient bio-reducing and stabilizing agents for nanoparticle formation [16,17].

The OBP, commonly referred to as Purple Basil, is distinguished by its complex and rich profile of bioactive compounds, which serve as the foundation for the development of effective phytocatalysts [15,18]. The plant’s distinct purple coloration is primarily attributed to a high concentration of anthocyanins, which coexist with a diverse array of phenolic acids and flavonoids [18,19]. These compounds are synthesized as secondary metabolites and are responsible for the potent antioxidant, anti-inflammatory, and antimicrobial properties exhibited by the extract [15,20]. Within this profile, the OBPE contains significant levels of total phenols that act as primary antioxidants by neutralizing the reactive oxygen species (ROS) through hydrogen atom donation [20]. Dominant phenolic acids, such as rosmarinic acid and chicoric acid, contribute to the high ferric reducing antioxidant power (FRAP) observed in the extract [20,21,22]. Furthermore, this variety is characterized by an abundance of flavonoids, including quercetin and eugenol derivatives [15,19], and these molecules possess hydroxyl groups that facilitate the reduction in metal ions during the synthesis of NPs by acting as both reducing and stabilizing agents [23,24]. As a distinctive trait of the purpurascens variety, anthocyanins provide not only the pigment but also enhance the radical scavenging capacity of the extract, as evidenced by 2,2-difenil-1-picrilhidrazil and 2,2′-Azino-bis (3-ethylbenzothiazoline-6-sulfonic acid) assays [15,18,25].

The implementation of OBPE in the synthesis of metal-oxide nanostructures, such as ZnO, leverages the “green chemistry” potential of these phytochemicals [26,27]. The functional groups identified via the Fourier Transform Infrared Spectroscopy (FTIR), specifically the C=O (carbonyl) from esters and the O–H (hydroxyl) from phenolic rings, play a crucial role in the nucleation and growth of inorganic crystals [28,29]. Consequently, the high total phenol content (measured at approximately 1807.28 ± 57.38 μmol GAE/g d.w.) and flavonoid content (33.17 ± 3.50 μmol QE/g d.w.) ensure that the extract provides sufficient electron density to stabilize the nanostructures [24,27]. Although subsequent thermal treatments may eliminate the organic phase to achieve the inorganic catalysts of high-purity, the initial interaction between the OBP phytochemicals and the metal precursors is essential for controlling the particle morphology and size distribution [28,29].

The synthesis of ZnO nanostructures mediated by OBPE relies on the synergistic action of its complex biochemical profile [12]. The primary mechanism involves the transformation of metal precursors into stable NPs through two distinct stages. Polyphenols and flavonoids present in the OBPE, characterized by a high concentration of total phenols (1807.28 ± 57.38 µmol GAE/g d.w.) [30], act as a dual complexing and structure-directing agent. The hydroxyl (–OH) groups attached to the aromatic rings of these molecules donate electrons to the Zn^2+^ ions [31,32]. This electron transfer facilitates the formation of zinc complexes, which, upon the addition of NaOH, subsequently initiate the nucleation process. FTIR analysis confirms this interaction through the observed vibrations of O–H stretching at 3230 cm^−1^ and aromatic C=C stretching at 1600 cm^−1^ [33].

Once nucleation occurs, the organic molecules in the extract function as capping agents. Compounds such as flavonoids and anthocyanins adsorb onto the surface of the growing ZnO nuclei through their carbonyl (C=O) and carboxylate groups, identified in the FTIR spectra near 1400 cm^−1^. These organic layers form a protective shell around the inorganic core that effectively lowers the surface free energy of the NPs. Additionally, the adsorbed phytochemicals provide steric hindrance and electrostatic repulsion, preventing the coalescence of particles [34]. This results in the spherical morphology and controlled size distributions observed in similar bio-assisted syntheses. The presence of these capping agents is further demonstrated by the retention of the bioactive signals in the infrared fingerprint, such as the C–O–C stretching at 1060 cm^−1^.

While the OBPE provides initial stability and morphology control, subsequent thermal treatments (TT) at temperatures between 700 °C and 900 °C ensure the complete elimination of any residual organic agents. The expected removal of this layer, confirmed by the disappearance of the C=O and –OH bands in the treated samples, yields high-purity crystalline ZnO while preserving the integrity of the nanostructure established during the green synthesis phase [35].

The incorporation of OBPE significantly alters the crystallization pathway of the metal oxides. Bioactive molecules, particularly flavonoids and polyphenols, lower the interfacial tension between the aqueous precursor and the forming solid phase [12,36]. This reduction in the energy barrier for nucleation allows for the formation of crystalline seeds at considerably lower temperatures than those required by conventional solid-state or purely inorganic solvothermal methods [36]. Through complexation and pre-organization, functional groups such as hydroxyls (–OH) and carbonyls (C=O) in the extract coordinate with Zn^2+^ ions to form intermediate complexes [31]. These complexes act as templates that direct site-specific growth and the spatial arrangement of atoms, promoting the development of specific crystalline planes, such as the (101) or (002) planes of the ZnO wurtzite structure [37]. Furthermore, the presence of the organic matrix provides kinetic control by limiting the diffusion rate of ions, which prevents uncontrolled crystal growth and favors the formation of nanometric crystalline domains even before the intensive thermal treatment [36,38].

Regarding the influence of the extract concentration on the crystalline integrity, evidence from dynamic light scattering and SEM analysis shows that the concentration of the extract directly dictates the final hydrodynamic diameter and, consequently, the crystalline grain size [36,39]. This involves synergistic stabilization where the successful encapsulation of bioactive compounds within a matrix, as observed in studies utilizing zein demonstrates how a controlled environment preserves the integrity of the system while maintaining the bioactivity of the sought-after compounds. While the pure extract provides the initial template, ensuring phase stability, the subsequent TT at temperatures such as 700 °C–900 °C is essential for achieving high crystallinity by removing residual amorphous organic matter and consolidating the inorganic lattice [40,41,42].

The ability of OBPE to induce crystallization at lower energetic thresholds is vital for the applications requiring sustained release and bioactivity. The preservation of high phenolic content (1807.28 ± 57.38 µmol GAE/g) and antioxidant capacity through the synthesis process ensures that the resulting nanostructures retain functional properties that complement the inorganic catalytic activity [17].

The landscape of ZnO nanoparticle synthesis has historically been dominated by chemical routes, most notably the direct precipitation and sol–gel methods. As reviewed by Kolodziejczak-Radzimska and Jesionowski [43], chemical precipitation utilizing strong bases like sodium hydroxide (NaOH) allows for high yields and a diverse array of morphologies, ranging from nanorods to complex flower-like structures. However, these processes often introduce environmental burdens due to the generation of hazardous chemical byproducts and the potential for residual ion contamination (e.g., Na+) on the particle surface. In contrast to this, the sol–gel method, further analyzed by Arya et al. [44] offers a more controlled path to crystallinity through the hydrolysis and condensation of metal alkoxides or salts. While sol–gel techniques provide high homogeneity, they remain dependent on the synthetic precursors and rigorous calcination protocols to achieve the desired hexagonal wurtzite phase, often necessitating the use of external surfactants to mitigate particle agglomeration.

Green synthesis has emerged as a sustainable alternative to conventional chemical routes for preparing ZnO nanomaterials, largely due to its reduced environmental impact, lower toxicity, and reliance on renewable biological resources [27]. In this approach, plant extracts function as the complex reaction media that simultaneously act as stabilizers and growth-directing agents during nanoparticle formation. These extracts typically contain a heterogeneous mixture of organic molecules, such as polyphenols, flavonoids, organic acids, sugars, and amino compounds, which interact with Zn^2+^ ions and influence the nucleation and crystal growth processes [45,46]. The chemical complexity of the plant extracts underpins many of the distinctive structural and functional features observed in green-synthesized ZnO when compared with materials produced by conventional chemical methods.

One of the principal advantages attributed to green-synthesized ZnO is the presence of plant-derived organic species on the nanoparticle surface, which can significantly influence the dispersion stability and surface chemistry. Adsorption of larger phytochemicals, particularly polyphenolic compounds, has been shown to suppress agglomeration and enhance the interfacial interactions with biological or polymeric matrices, thereby improving the material’s suitability for the applications requiring biocompatibility, antimicrobial activity, or surface functionalization [47,48]. In addition, the organic coating formed during green synthesis may contribute to improved optical and biological responses compared with the ZnO produced via purely chemical routes [49].

Despite these advantages, significant challenges remain regarding the structural purity, reproducibility, and precise control of material properties in green-synthesized ZnO. The intrinsic variability in the plant extract composition can lead to differences in crystallinity, defect density, and optical response, even when the nominally identical synthesis conditions are employed [43,46]. While numerous studies report enhanced functional performance of green-synthesized ZnO, comparatively fewer investigations systematically correlate the extract chemistry, synthesis parameters, and post-treatment conditions with intrinsic structural features. This lack of mechanistic understanding constitutes an important research gap, particularly in distinguishing the surface-related effects from bulk structural modifications.

Within this context, increasing attention has been directed toward the debated role of phytochemicals in influencing ZnO defect chemistry and lattice-related properties. During phyto-mediated synthesis, small organic molecules or decomposition fragments present in the extract may transiently interact with ZnO nuclei during the early stages of crystallization, potentially affecting defect formation pathways or inducing a local lattice disorder [50,51]. While larger phytochemicals predominantly remain adsorbed on the nanoparticle surface and act as the capping agents, smaller carbon- or nitrogen-containing species may indirectly influence the electronic and optical properties through the defect-related mechanisms rather than through the direct substitution into the ZnO lattice [11,29].

Post-synthesis thermal treatment further modulates this behavior. Annealing can promote the decomposition of organic residues, facilitate the formation or redistribution of intrinsic defects such as oxygen vacancies and zinc interstitials, and alter charge carrier dynamics within the semiconductor [44]. These defect-mediated changes are often reflected in the shifts in band gap energy, variations in photoluminescence behavior, and enhanced visible-light absorption, which are commonly used to distinguish green-synthesized ZnO from chemically synthesized analogs [52]. Consequently, rather than acting as classical substitutional dopants, phytochemicals in green synthesis are more accurately understood as contributors to surface functionalization and defect modulation, highlighting the complex interplay between the synthesis conditions, organic components, and the resulting physicochemical properties of ZnO nanomaterials.

Motivated by the specific phytochemical potential of OBP, this study reports a facile green synthesis route to produce ZnO NPs. The research investigates the capability of OBPE to act as a dual precipitating and structure-directing agent under mild reaction conditions, without the immediate application of high-temperature calcination. To comprehensively elucidate the physicochemical quality of the material, a systematic characterization was conducted using UV-Visible (UV-Vis), FTIR, XRD, Energy Dispersive X-ray Spectroscopy (EDS), and SEM techniques. The investigation focuses on tracking the structural and morphological evolution of the NPs from their as-synthesized state through a thermal treatment range of 700 °C to 900 °C. By correlating the behavior of the organic capping agents with the lattice consolidation and microstructural growth, this work aims to validate a sustainable methodology for producing tunable ZnO semiconductors suitable for optoelectronic and biomedical applications.

## 2. Materials and Methods

### 2.1. Materials

Zinc acetate dihydrate (Zn(CH_3_COO)_2_·2H_2_O, ACS grade; Fermont, Monterrey, Mexico) and sodium hydroxide (NaOH, ACS grade; Fermont, Monterrey, Mexico) were used as chemical precursors without further purification. Absolute ethanol (ACS grade) and deionized water were utilized for the preparation of the *OBPE* and the subsequent synthesis and washing of the NPs.

### 2.2. Preparation of OBPE

The OBP leaves were obtained as reported by Hernández-Abril (2024) [17]. The OBPE was prepared by pulverizing the desiccated leaves into a fine powder, followed by magnetic stirring in a 1:1 (*v*/*v*) ethanol:water solution for 15 min at room temperature. The obtained mixture was subjected to ultrasonic treatment for 5 min to enhance the extraction. Subsequently, the suspension was filtered using standard filter paper, and the filtrate was stored under refrigeration for further experimental use.

### 2.3. Experimental Design and Green Synthesis

This study was structured as a controlled univariate experimental design to precisely isolate the thermodynamic effects of calcination on the structural and optical properties of the NPs. The concentration of the OBPE and the initial precursor volume ratio (10 mL of OBPE added to 250 mL of a 20 mM zinc acetate solution) were kept strictly constant across all experimental units. Therefore, the sole independent variable evaluated was the thermal treatment temperature (as-synthesized, 700 °C, 800 °C, and 900 °C). To ensure macroscopic reproducibility and batch-to-batch consistency of the phyto-mediated synthesis, the precipitation process was standardized under identical laboratory conditions. The mixture was subjected to continuous magnetic stirring for 30 min, after which the pH was adjusted to 12 by the dropwise addition of 1 M NaOH. The reaction was maintained under constant magnetic stirring for an additional 2.5 h. ZnO was synthesized via the following reaction:

Dissociation of the precursor and complexation with OBPE phytochemicals:

Zn(CH_3_COO)_2_ + OBPE → [Zn^2+^(OBPE)] (1)

Formation of zincate species (at pH 12):

[Zn^2+^(OBPE)] + 4(OH)^−1^ + [Zn(OH)_4_]^2−^ (2)

Dehydration and nucleation of ZnO:

[Zn(OH)_4_]^2−^ ↔ ZnO (3)

**Figure 1:**
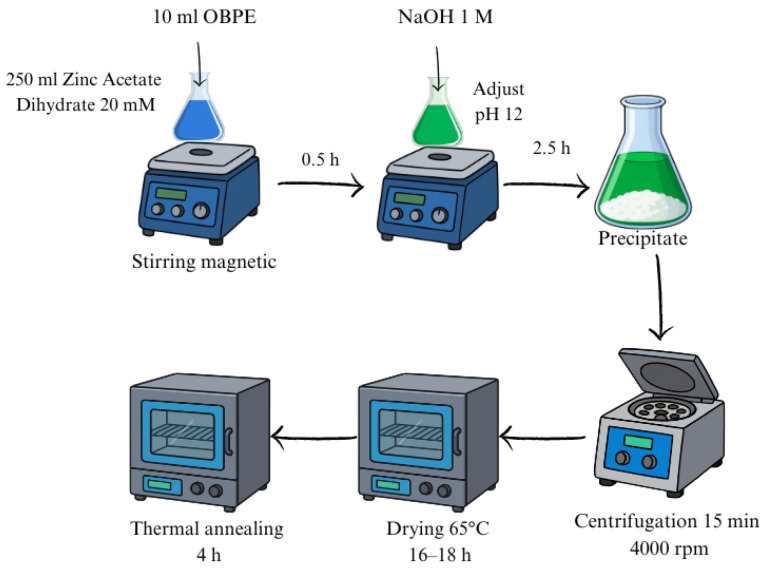
Schematic representation of the phyto-mediated green synthesis of ZnO NPs using OBPE.

The resulting precipitate was washed with deionized water and isolated via centrifugation at 4000 rpm for 15 min. Finally, the obtained material was dried in an air atmosphere at 65 °C for 18–19 h. A schematic representation of the initial synthesis procedure is illustrated in Figure 1.

To evaluate the structural and optical evolution of the material, portions of the as-synthesized powder were subsequently subjected to thermal treatment in a conventional oven (ARSA Model AR-340; Fabricantes Feligneo, S. A. de C.V, Zapopan, Jalisco, Mexico) under an air atmosphere. The specific annealing parameters and the designated nomenclature for each resulting sample are detailed in Table 1.

### 2.4. Optical Characterization (UV-Vis and Diffuse Reflectance)

To determine the absorption spectra, the synthesized materials were first suspended in deionized water and sonicated for 3 min to prevent agglomeration. The resulting homogeneous suspensions were then analyzed in quartz cuvettes using a VE-5100UV spectrophotometer (VELAB Co., Pharr, TX, USA). Baseline adjustments were systematically applied using a deionized water blank. Additionally, to evaluate the optical band gap (Eg) of the solid-state materials, diffuse reflectance measurements of the ZnO powders were carried out using a PerkinElmer Lambda 365 UV/Vis spectrophotometer (PerkinElmer, Waltham, MA, USA).

### 2.5. FTIR

The functional groups present on the nanoparticle surface and the thermal degradation of organic components were analyzed using a Nicolet iS50 FT-IR spectrophotometer (Thermo Fisher Scientific, Waltham, MA, USA). The infrared spectra of the powdered samples were recorded in the wavenumber range of 4000 cm^−1^ to 500 cm^−1^.

### 2.6. X-Ray Diffraction (XRD)

The crystallographic structure, phase purity, and crystallite size of the samples were analyzed using a Bruker AXS D8 Advance diffractometer (Bruker AXS, Karlsruhe, Germany) equipped with a CuKα radiation source. Data acquisition was carried out at room temperature in the 2θ angular range from 20° to 70°.

### 2.7. Scanning Electron Microscopy (SEM) and Statistical Analysis

The surface morphology and elemental composition (via EDS) were evaluated using a JEOL JSM-7800F scanning electron microscope (JEOL Ltd., Tokyo, Japan) operating at an accelerating voltage of 15 kV. Prior to analysis, the powdered samples were mounted onto aluminum stubs using double-sided carbon-conductive tape. To evaluate the influence of the thermal treatment temperature on particle size evolution, a rigorous statistical analysis was performed by measuring over 400 individual particles (N ≥ 400) per sample from random fields of view. A one-way analysis of variance (ANOVA) was conducted on the dataset, with differences considered statistically significant at a *p*-value < 0.05. Post hoc pairwise comparisons were subsequently performed using Tukey’s Honest Significant Difference (HSD) test to identify specific variations and group samples with statistically comparable means.

## 3. Results and Discussion

### 3.1. Phase Purity and Crystallite Growth Dynamics

**Figure 2:**
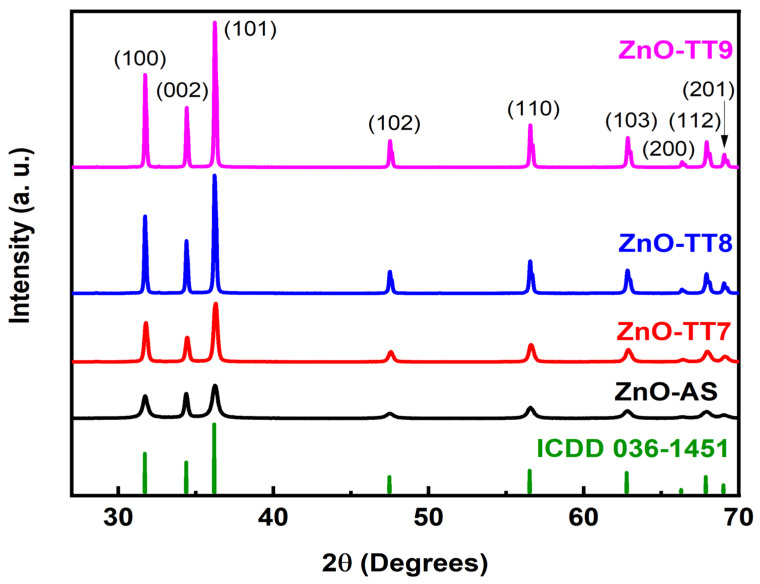
XRD patterns of the ZnO nanoparticles synthesized using OBPE. All of the diffraction peaks match the standard hexagonal ZnO phase with high precision according to the ICDD card no. 036-1451. Notably, no characteristic peaks of impurities or secondary phases were detected as the calcination temperature increased from 300 °C to 500 °C, confirming high crystalline purity. The progressive narrowing of the (101) reflection peak indicates an increase in the average crystallite size, calculated via the Scherrer equation.

Figure 2 displays the XRD patterns of the ZnO NPs, both as-synthesized and calcined at 700 °C, 800 °C, and 900 °C for 4 h. At the bottom, the reference XRD pattern exhibits diffraction peaks corresponding exclusively to the hexagonal ZnO wurtzite structure, matching the samples with high precision (ICDD 036-1451). There are no additional diffraction peaks to indicate the presence of impurities or secondary phases, confirming the high phase purity achieved by this phyto-mediated synthesis method.

**Figure 3:**
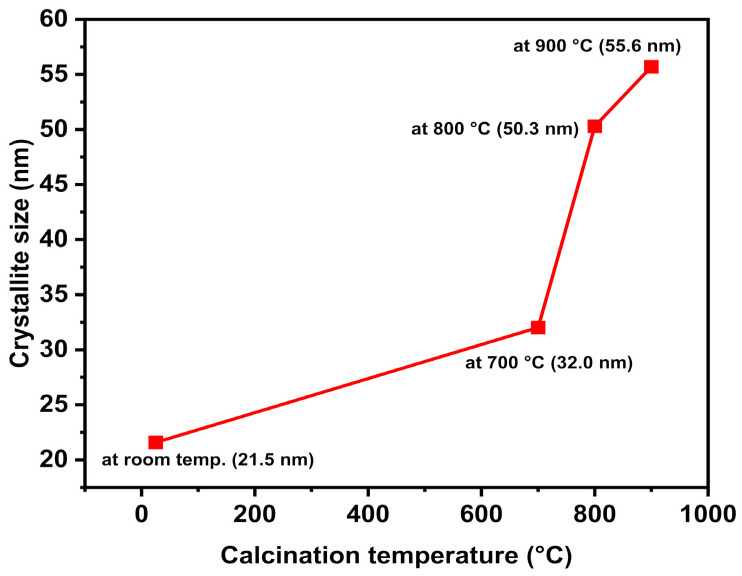
Correlation between calcination temperature and the average crystallite size of the ZnO nanoparticles. The results demonstrate a monotonic increase in crystallite size, indicating that higher thermal energy promotes a systematic coalescence of nanocrystals. This trend reflects the progressive improvement of the crystalline order, resulting in a more defined hexagonal wurtzite structure.

Scherrer’s formula was used to calculate the average crystallite sizes of the samples, utilizing the full width at half maximum (FWHM) of the most intense (101) XRD peaks. As shown in Figure 3, the trend of the crystallite sizes is consistent with standard thermodynamic behavior, increasing proportionally from 21.5 nm for the ZnO-AS sample to 32.0 nm, 50.3 nm, and 55.6 nm for the samples calcined at 700 °C, 800 °C, and 900 °C, respectively [44,53,54]. The ZnO-AS sample exhibits the smallest crystallite size; this restriction is primarily due to the adsorbed organic layer of the OBPE, which acts as a physical barrier that limits the ionic diffusion and crystallite growth during precipitation. Upon applying calcination temperatures from 700 °C to 900 °C, the thermal degradation of these organic residues eliminates the physical barrier, allowing the individual crystallites to coalesce and form larger polycrystalline grains, promoting an improvement in crystalline quality and a reduction in the intrinsic lattice defects.

Consequently, the high phase purity of the ZnO-AS sample represents a highly significant finding in the XRD analysis. Although it exhibits lower diffraction intensities and slightly broader peaks compared to the calcined samples, indicative of its smaller crystallite size (21.5 nm), it achieved a pure crystalline structure without requiring an initial calcination step. This result provides a distinct advantage over the conventional synthesis routes reported in the literature, where ZnO NPs typically mandate thermal annealing to achieve the acceptable crystallinity and higher intensity. For instance, Omri et al. [55] synthesized ZnO NPs with a hexagonal wurtzite structure via a conventional sol–gel method. Their study aligns with the literature, showing that increasing the calcination temperature from 300 °C to 750 °C increases the average grain size from 33 nm to 52 nm. However, the XRD of their as-prepared sample showed significant impurities around 2θ = 40°–45°, requiring thermal treatment to reach higher purity.

Similarly, Kayani et al. [41] reported the necessity of a calcination process to improve the quality of ZnO NPs, as impurity peaks appeared in their uncalcined reference sample at 2θ = 50°–55° and 60°. Furthermore, their crystallite size increased from 27.9 nm to 34.9 nm as the temperature was raised from room temperature to 750 °C. In a related study, Mornani et al. [56] synthesized ZnO NPs using zinc nitrate hexahydrate, ethylene glycol, and hydrazine. Their XRD analysis revealed that prior to the calcination process, there were numerous impurities and contaminants in the sample; only after applying the calcination temperatures from 400 °C to 650 °C did the samples exhibit a pure hexagonal wurtzite structure. Additionally, they reported an anomalous decrease in the crystallite size from 66 nm to 46 nm as the temperature increased from 400 °C to 650 °C, a trend that remains unusual in the literature and lacks a physical explanation by the authors. In contrast, the OBPE-mediated synthesis presented in this study successfully bypasses these intermediate impurity phases, yielding phase-pure wurtzite ZnO directly from the initial precipitation.

### 3.2. Morphological and Elemental Composition Analysis (SEM-EDS)

**Figure 4:**
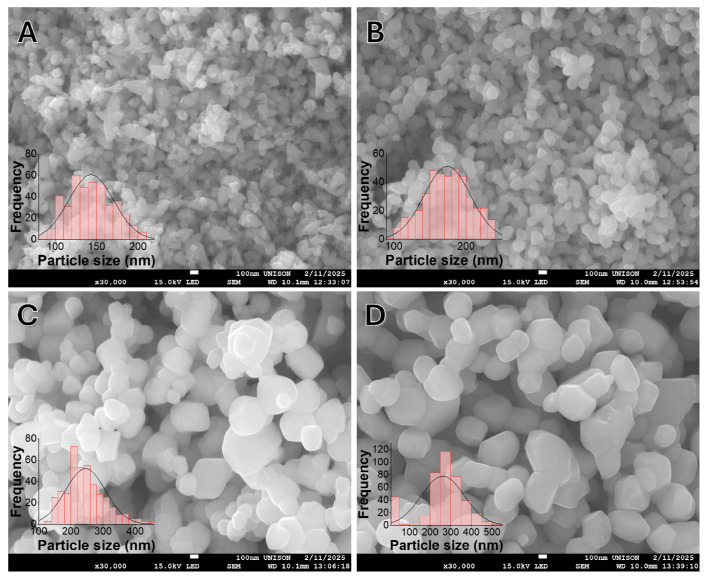
SEM micrographs and corresponding particle size distribution histograms of (**A**) ZnO-AS, (**B**) ZnO-TT7, (**C**) ZnO-TT8, and (**D**) ZnO-TT9 NPs. The images reveal a transition from small, quasi-spherical nuclei in the as-synthesized sample to larger, well-defined hexagonal-like structures as the calcination temperature increases. The histograms demonstrate a systematic shift in the mean particle size, confirming that higher thermal treatment promotes grain growth and decreases the dispersion of the distribution. This morphological maturation is consistent with the enhanced crystalline order observed in the XRD analysis.

The surface morphology and particle size distribution of the ZnO NPs were evaluated using SEM. Figure 4 reveals the morphological evolution of NPs as a function of thermal treatment. The as-synthesized sample (ZnO-AS, Figure 4A) exhibits a highly agglomerated structure with an irregular morphology. As the calcination temperature increases to 700 °C, 800 °C, and 900 °C (Figure 4B, Figure 4C, and Figure 4D, respectively), the particles undergo a distinct morphological transition, coalescing to form larger, well-defined polyhedral and quasi-hexagonal shapes characteristic of the wurtzite structure [43,44].

The embedded histograms demonstrate a continuous increase in the average particle size, shifting from 143.33 nm for the uncalcined sample to 261.50 nm for the sample treated at 900 °C. Statistical analysis confirms that the variation in particle size between each thermal treatment is significantly different (denoted by distinct superscript letters in Table 2). It is crucial to note that the average particle sizes observed via SEM are substantially larger than the crystallite sizes calculated from the XRD data (21.5 nm to 55.6 nm). This disparity indicates that the microscopic particles observed in SEM are in fact polycrystalline agglomerates. Fundamentally, this polycrystalline nature is already established during the initial precipitation process, where several smaller nanometric crystallites cluster together to form larger aggregates. Subsequently, the application of high temperatures further pronounces this agglomeration, driving the continued fusion and growth of these microstructures through Ostwald ripening [57].

To confirm the chemical nature of these microstructures and evaluate the influence of the OBPE on the purity of the final product, elemental composition analysis via EDS was performed. The quantitative results are summarized in Table 2.

Within the ZnO-AS sample, the presence of zinc and oxygen was identified as expected. For this uncalcined sample, the observed mass composition was 82.73% zinc and 17.27% oxygen. This proportion is remarkably close to the ideal ZnO stoichiometry, demonstrating that the green synthesis route mediated by OBPE facilitates the spontaneous and highly efficient formation of the metal oxide during the initial precipitation process, unlike traditional sol–gel methods that strictly require calcination to eliminate the chemical precursors [44]. The phytocompounds present in the extract act as structure-directing agents that promote ZnO crystallization at room temperature prior to any thermal treatment [58].

The slight deviation from the ideal value in the ZnO-AS sample can be attributed to the presence of a residual organic layer originating from the OBPE, which manifests in the EDS spectrum as a carbon signal in the low-energy region [29,59]. This observation establishes a direct correlation with the surface chemistry analysis, where these functional groups act as capping agents to stabilize the nanoparticle surface, as will be fully corroborated by the FTIR analysis.

A comparison of these results with the literature underscores the advantage of using OBPE. While other studies require calcination to eliminate the intermediate impurity phases [29,59], the EDS analysis of the ZnO-AS sample provides evidence of high elemental purity immediately following the 65 °C drying stage. However, it is important to address the sub-stoichiometric oxygen content observed across the samples. Theoretical pure ZnO consists of approximately 80.34 wt% Zn and 19.66 wt% O. The generally lower oxygen mass percentages observed herein are indicative of an intrinsic non-stoichiometric structure, widely reported in the chemically synthesized ZnO, which is typically dominated by the oxygen vacancies of V_O_ and Zn_i_.

The evolution of the elemental composition in Table 2 reflects a clear thermal dynamic. The initial oxygen content in the ZnO-AS sample (17.27 wt%) is partially influenced by the presence of the residual organic capping layer from the OBPE. Upon calcination, the thermal degradation of this organic layer and the removal of water are completed. However, the material remains inherently oxygen-deficient even at the highest annealing temperatures (e.g., reaching a normalized oxygen mass of 18.22 wt% in ZnO-TT9, still below the 19.66 wt% theoretical value). As the annealing temperature increases, thermal energy intrinsically promotes the creation of the oxygen vacancies within the lattice rather than filling them. This phenomenon dictates that as the temperature rises, the mass of ZnO slightly decreases due to the continuous generation of the V_O_ [60,61]. Despite this fundamental non-stoichiometric nature, this capacity of the OBPE to facilitate structural consolidation from the initial stages of synthesis establishes this method as a highly efficient alternative compared to the other natural stabilizing agents [62].

### 3.3. FTIR and Surface Chemistry

**Figure 5:**
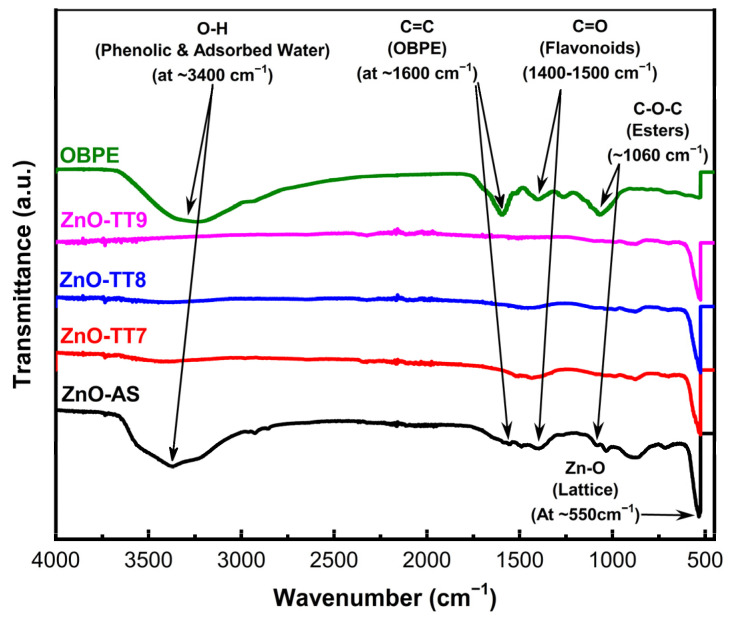
FTIR spectra of as-synthesized (ZnO-AS) and calcined (ZnO-TT7, ZnO-TT8, ZnO-TT9) ZnO nanoparticles. The ZnO-AS sample reveals intense bands associated with the OBPE organic matrix, including phenolic O–H and adsorbed water (~3400 cm^−1^), aromatic C=C and water bending (~1600 cm^−1^), carbonyl C=O (1400–1550 cm^−1^), and ester C–O–C (1060 cm^−1^), identifying them as surface organic agents. The successful formation of the crystalline lattice of the Zn-O at 450–550 cm^−1^ in samples from ZnO-TT7 to ZnO-TT9 is accompanied by the disappearance of the organic signals.

Figure 5 displays the FTIR spectra of the as-synthesized ZnO (ZnO-AS) and the samples subjected to various calcination temperatures (700 °C, 800 °C, and 900 °C). The most prominent and fundamental feature across all spectra is the sharp, intense absorption band in the 450 cm^−1^–550 cm^−1^ region. This peak corresponds to the characteristic Zn–O stretching vibration in the tetrahedral sites of the wurtzite structure [63], confirming the successful formation of the ZnO crystalline lattice from the initial precipitation stage.

In the high-frequency range (3200 cm^−1^–3500 cm^−1^), the as-synthesized sample (ZnO-AS, black line) exhibits a broad band centered at approximately 3400 cm^−1^. While this region typically includes the contributions from the adsorbed moisture, in the context of this phyto-mediated synthesis, it is primarily assigned to the O–H stretching modes of the phenolic and alcoholic compounds originating from the OBPE. Similarly, the signal observed at approximately 1600 cm^−1^ represents an overlap between the H–O–H bending vibration of water and the aromatic C=C stretching modes from the phenolic rings in the extract.

A crucial finding in the ZnO-AS spectrum is the presence of signals between 1400 cm^−1^ and 1550 cm^−1^ (C=O stretching) and a distinct shoulder at 1060 cm^−1^ (C–O–C stretching). These functional groups identify the organic molecules, specifically flavonoids, phenolic acids, and anthocyanins, acting as a stabilizing organic layer. These agents encapsulate the ZnO nuclei, providing a steric hindrance and an electrostatic repulsion that prevent particle coalescence and control the final morphology [64,65].

As the calcination temperature increases to 900 °C, these organic-related bands undergo a drastic reduction and eventual disappearance. This signifies the effective thermal degradation of the organic layer and the dehydration of the samples, yielding a high-purity inorganic phase [66,67]. This chemical refinement directly correlates with the stoichiometric purification observed in the EDS analysis, where the sample approaches the theoretical oxygen mass of the pure wurtzite ZnO as the carbonaceous components are removed.

### 3.4. UV-Vis Spectroscopic Analysis

**Figure 6:**
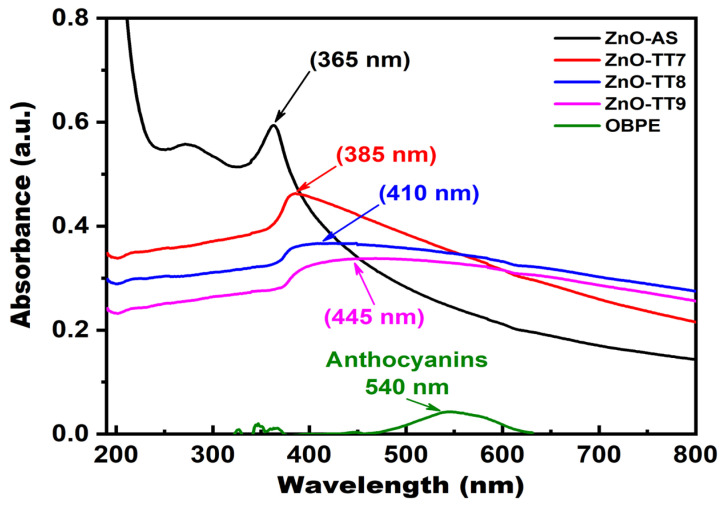
UV-Vis absorption spectra of the as-synthesized precursor, the calcined samples (700 °C, 800 °C, and 900 °C) of ZnO NPs, and the raw OBPE. The spectra exhibit a prominent redshift in the absorption edge as the calcination temperature increases, moving from the UV toward the visible region. This red-shift is attributed to the relaxation of the quantum confinement effects and the reduction in the bandgap as the crystalline structure matures. Additionally, the disappearance of the characteristic organic absorption bands from the OBPE in the calcined samples confirms the complete thermal degradation of the phytochemical templates and the formation of high-purity ZnO.

For the UV-Vis spectra shown in Figure 6, the as-synthesized ZnO NPs (black line) exhibit a characteristic excitonic absorption peak at approximately 365 nm. This peak corresponds to the intrinsic electronic transition from the valence band to the conduction band (O 2p to Zn 3d), which serves as a strong indicator of the successful formation of the crystalline ZnO phase without the initial requirement of thermal treatment [68,69]. This specific peak position is in good agreement with the optical signatures reported for nanostructured ZnO obtained via other green synthesis methodologies [70,71].

Analysis of the OBPE (green line) highlights the role of the secondary metabolites of the plant in the reaction. The extract shows distinct absorption bands corresponding to phenolic acids such as rosmarinic and chicoric acids and a broad band in the visible range (approximately 540 nm) characteristic of anthocyanins [19,72,73]. In the nanoparticle samples (Figure 6), the distinct attenuation of these phytochemical signatures indicates that the organic molecules transitioned from a free state to act as chelating and capping agents, actively coordinating with the zinc ions during the nucleation and stabilization of the ZnO lattice [70].

The influence of thermal treatment is shown in Figure 6, where decreased absorption intensities are observed in the samples calcined at 700 °C, 800 °C, and 900 °C (red, blue, and pink lines, respectively). This overall decrease in absorption intensity is primarily attributed to the significant increase in particle size and agglomeration induced by the calcination process, as corroborated by the SEM analysis. Larger, denser microparticles exhibit increased light scattering and faster settling rates while in suspension during the measurement, resulting in a lower recorded absorbance compared to the smaller ZnO-AS nanoparticles, which remain stabilized by an adsorbed organic layer [68,71]. Furthermore, the observed red-shift in the absorption edge in the calcined samples indicates that the NPs underwent agglomeration or crystal growth due to the thermal energy applied [74,75]. Moreover, the appearance of the fundamental ZnO excitonic peak in the as-synthesized sample validates the efficiency of the OBPE in facilitating crystallization, offering a significant thermodynamic advantage over traditional synthesis routes [69,75].

### 3.5. Diffuse Reflectance Spectroscopy and Eg Analysis

**Figure 7:**
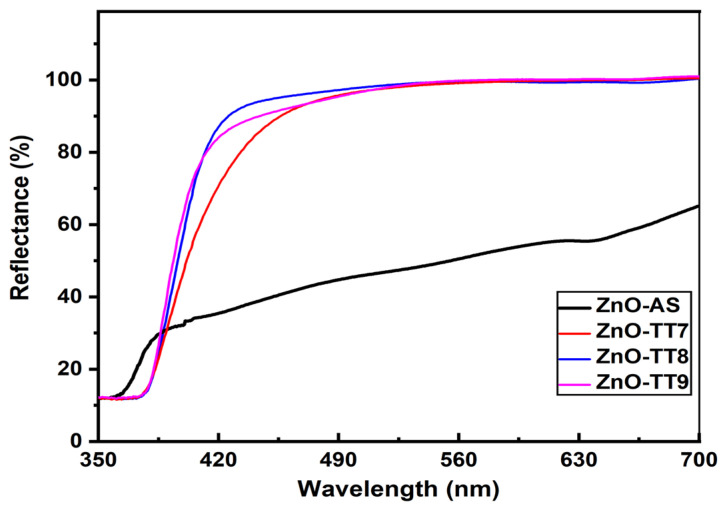
Raw diffuse reflectance spectra of the as-synthesized (ZnO-AS) and calcined (ZnO-TT7, ZnO-TT8, and ZnO-TT9) ZnO NPs. The as-synthesized material exhibits significantly lower reflectance across the visible spectrum compared to the calcined samples; this optical behavior is attributed to the intense light absorption by residual organic phytochemicals from the OBPE template. Upon thermal treatment, the marked increase in reflectance and the emergence of a sharp absorption edge confirm the effective removal of organic matter and the consolidation of the high-purity ZnO crystalline phase.

The optical properties and the electronic structure of the synthesized ZnO NPs were further investigated using diffuse reflectance spectroscopy. The raw reflectance spectra (Figure 7) demonstrate that all samples exhibit high absorption (low reflectance) in the ultraviolet (UV) region, characteristic of the intrinsic band-to-band transition of the ZnO semiconductor [76]. A crucial observation is that the as-synthesized sample (ZnO-AS) displays significantly lower reflectance (ranging from 30% to 60%) across the visible spectrum (400 nm–700 nm) compared to the thermally treated samples. This specific optical behavior, clearly visible in Figure 7, is directly attributed to the presence of residual organic agents and phytochemicals from the OBPE, such as anthocyanins and phenolic acids, which act as capping agents on the NPs after the 65 °C drying stage. Macroscopically, this optical absorption is consistent with the visual appearance of the synthesized powders; the ZnO-AS sample retains a darker, localized pigmentation due to the organic matrix, whereas the calcined samples (from ZnO-TT7 to ZnO-TT9) exhibit the stark white coloration characteristic of highly pure, inorganic ZnO. Upon thermal treatment between 700 °C and 900 °C, the reflectance in the visible region increases dramatically to roughly 95–100%. This increase in transparency correlates with the effective removal of the residual capping agents and the dehydration of the samples, a phenomenon previously evidenced by the disappearance of the C=O and –OH bands in the FTIR analysis. Furthermore, a noticeable red-shift in the absorption edge is observed in Figure 7 for all of the calcined samples compared to the ZnO-AS sample, indicating an improvement in the crystalline quality and a reduction in the intrinsic lattice defects driven by the applied thermal energy.

**Figure 8:**
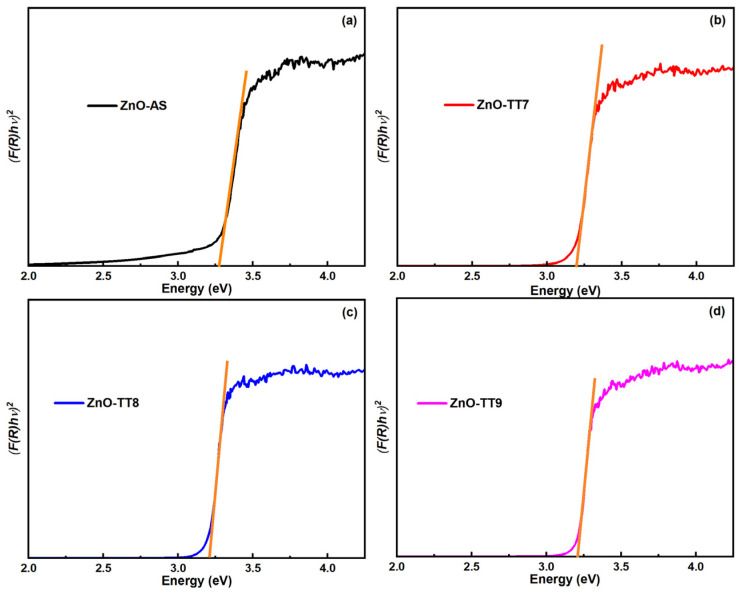
Tauc plots of [*F*(*R*)*hν*]^2^ versus photon energy (*hν*) derived from diffuse reflectance data for (**a**) ZnO-AS, (**b**) ZnO-TT7, (**c**) ZnO-TT8, and (**d**) ZnO-TT9. The graphs illustrate the linear extrapolation (orange line) of the absorption edge to the energy axis, used to determine Eg. A systematic decrease in the Eg values is observed as the calcination temperature increases, reflecting the transition from a quantum-confined regime to a bulk-like crystalline state. This trend is consistent with the structural maturation and grain growth identified in the XRD and SEM analyses.

To quantify these changes, Eg was determined by applying the Kubelka-Munk function to the diffuse reflectance data and constructing the respective Tauc plots (Figure 8). The constructed plots facilitate the linear extrapolation to [*F*(*R*)*hν*]^2^ = 0 to find Eg. The calculated Eg values for all samples are lower than the theoretical value of 3.37 eV typically reported for bulk ZnO [74,77]. The ZnO-AS sample exhibits the widest Eg among the set (approximately 3.28 eV), which is consistent with its smaller crystallite size as determined by XRD [78]. This relative widening of the Eg is primarily attributed to quantum confinement effects resulting from the confined crystallite size of the uncalcined material [79], as well as the lattice strain induced by the surface-adsorbed organic compounds [80]. In contrast, a significant red-shift is observed following the thermal treatments, where the Eg values drop and stabilize at approximately 3.21 eV for all calcined samples (ZnO-TT7, ZnO-TT8, and ZnO-TT9). This value falls precisely within the reported Eg ranges for synthesized ZnO NPs [57,81] and aligns with the red-shift observed in the original UV-Vis absorption data. The identical Eg values obtained across the calcination range suggest that once the organic phase is successfully removed and the initial lattice consolidation occurs at 700 °C, the fundamental electronic structure of the semiconductor reaches a stable state, regardless of further increases in the crystallite size (up to 55.6 nm) [82].

Although the thermal annealing of the structural defects (such as oxygen vacancies) is typically expected to widen the Eg toward the bulk ZnO value of 3.37 eV, a continuous red-shift is observed. This phenomenon is primarily attributed to the dominant role of residual strain and morphological evolution. As the NPs coalesce and undergo grain growth at elevated temperatures, the accumulated tensile microstrain within the wurtzite lattice alters the electronic band structure, effectively lowering the conduction band edge. This strain-induced narrowing, combined with the loss of quantum confinement, prevents the expected recovery to the bulk Eg value [41].

To provide a comprehensive overview of the interrelated physical properties discussed, the calculated optical parameters, along with the structural data obtained from the XRD analysis, are summarized in Table 3.

This consolidated view highlights that while the crystallite size increases significantly with temperature, the band gap energy remains constant at 3.21 eV after the initial calcination. This behavior confirms that the OBPE-mediated synthesis allows for the precise tuning of the ZnO electronic structure, reaching a stable semiconducting state once the organic capping phase is removed. This conclusion is highly consistent with the stoichiometric refinement and high elemental purity corroborated by the EDS results.

## 4. Conclusions

This study validates the efficacy of OBPE as a highly efficient natural complexing and stabilizing agent for the synthesis of ZnO NPs. Unlike conventional physicochemical routes, this green method enabled the attainment of the crystalline hexagonal wurtzite phase with high elemental purity immediately following the initial synthesis stage, effectively eliminating the critical dependence on high-temperature annealing process for phase formation. Thermal evolution analysis demonstrated that the increase in the treatment temperature from 700 °C to 900 °C promotes a proportional growth in both the crystallite size (from 21.5 nm to 55.6 nm) and particle size (from 143.33 nm to 261.50 nm) through sintering and Ostwald ripening mechanisms. Furthermore, FTIR spectroscopy and EDS analysis confirmed the progressive degradation of organic superficial agents—such as polyphenols and anthocyanins—resulting in a stoichiometric refinement that closely approaches the theoretical purity of the wurtzite phase (18.22% normalized oxygen mass) at 900 °C. The optical tunability of the material was also established, evidenced by a red-shift in the band gap energy from 3.28 eV to 3.21 eV and a drastic increase in the visible diffuse reflectance upon the removal of the organic phase. In conclusion, the utilization of Purple Basil represents a sustainable, energy-efficient alternative that offers a thermodynamic advantage for the potential industrial-scale production of ZnO semiconductors. Future studies should explore the integration of these tunable nanomaterials into specific functional devices, such as high-efficiency photocatalytic systems or biocompatible sensors, to fully leverage their phyto-mediated properties.

## Figures and Tables

**Table 1:** Sample identification and corresponding thermal treatment conditions.

Sample ID	Treatment Temperature (°C)	Time (h)
ZnO-AS	As-synthesized (No calcination)	-
ZnO-TT7	700	4
ZnO-TT8	800	4
ZnO-TT9	900	4

**Table 2:** Average particle size and normalized elemental composition of the synthesized ZnO NPs.

Sample ID	Treatment Temp. (°C)	Average Particle Size (nm)	Mass Norm. Zn [%]	Mass Norm. O [%]	Atomic Zn [%]	Atomic O [%]
ZnO-AS	As-synthesized	143.33 ± 27.18 ^a^	82.73	17.27	53.97	46.03
ZnO-TT7	700	174.09 ± 31.98 ^b^	87.33	12.67	62.78	37.22
ZnO-TT8	800	245.12 ± 62.63 ^c^	85.96	14.04	59.97	40.03
ZnO-TT9	900	261.50 ± 103.99 ^d^	81.78	18.22	52.34	47.66

Different superscript letters indicate statistically significant differences between the particle sizes (*p* < 0.05).

**Table 3:** Summary of structural and optical parameters of ZnO NPs at different thermal treatments.

Sample ID	Treatment Temperature (°C)	Crystallite Size (D, nm)	Eg (eV)
ZnO-AS	As-synthesized	21.5	3.28
ZnO-TT7	700	32.0	3.21
ZnO-TT8	800	50.3	3.21
ZnO-TT9	900	55.6	3.21

## Data Availability

The data presented in this study are available on request from the corresponding author.

## Abbreviations

The following abbreviations are used in this manuscript:

ANOVA	Analysis of variance
EDS	Energy Dispersive X-ray Spectroscopy
FDA	Food and Drug Administration
Basil	*Ocimum basilicum*
Purple Basil	Purpurascens variety
FRAP	Ferric reducing antioxidant power
FTIR	Fourier Transform Infrared
FWHM	Full width at half maximum
GRAS	Generally Recognized As Safe
HSD	Honest Significant Difference
NPs	Nanoparticles
OBP	*Ocimum basilicum* var. *purpurascens*
OBPE	*Ocimum basilicum* var. *purpurascens* extract
ROS	Reactive oxygen species
SEM	Scanning electron microscopy
TT	Thermal treatments
UV	Ultraviolet
UV-Vis	UV-Visible
XRD	X-ray diffraction
ZnO	Zinc oxide
Eg	Optical band gap
GAE	Gallic Acid Equivalent
QE	Quercetin Equivalent
